# A Recalcitrant Electrical Storm and Implantable Defibrillator Exhaustion: Treatment Implications According to and Beyond Guidelines

**DOI:** 10.1016/j.jaccas.2019.09.016

**Published:** 2019-10-23

**Authors:** Christos Kontogiannis, Georgios Georgiopoulos, Christos Papageorgiou, Hector Anninos, Konstantinos Tampakis, Marinos Kosmopoulos, Panagiotis Vasileiou, Ioannis Kanakakis, Ioannis Paraskevaidis, Sofia Chatzidou

**Affiliations:** Department of Clinical Therapeutics, “Alexandra” Hospital, School of Medicine, National and Kapodistrian University of Athens, Athens, Greece

**Keywords:** beta-blocker, electrical storm, implantable cardioverter defibrillator, ventricular arrhythmia, ES, electrical storm, HF, heart failure, ICD, implantable cardioverter-defibrillator, ICU, intensive care unit, IV, intravenous, VF, ventricular fibrillation, VT, ventricular tachycardia

## Abstract

A 60-year-old patient presented with recalcitrant electrical storm (ES). Mild sedation and initial antiarrhythmic combination of esmolol and amiodarone did not affect the intensity of ES, which resulted in battery exhaustion. Oral propranolol in addition to intravenous amiodarone might be preferred in hemodynamically stable patients before interventional therapies. (**Level of Difficulty: Intermediate.**)

## Graphical abstract

**Figure:**
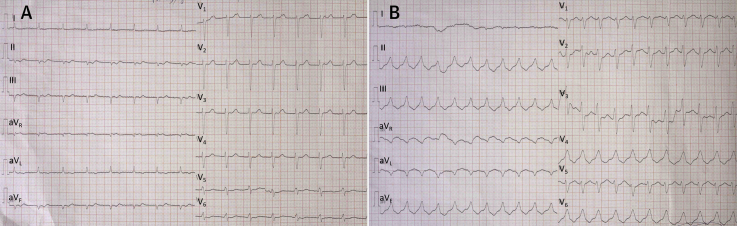

Approximately 30% of patients with implantable cardioverter-defibrillator (ICDs) will experience an episode of electrical storm (ES), which is characterized by ≥3 recurrences of ventricular arrhythmias within a 24-h period that activate appropriate device interventions (1). ES is a common problem in patients with heart failure (HF) treated with ICD implantation for secondary prevention of sudden cardiac death and is associated with an unfavorable prognosis (2). However, its management remains controversial because the selection of appropriate therapeutic regimens has not been deciphered by randomized clinical studies. The authors present a case of recalcitrant ES with incessant episodes of ventricular tachycardia/ventricular fibrillation (VT/VF) that resulted in exhaustion of the ICD’s battery after numerous shocks.

**Learning Objectives**

• To be able to select the optimal treatment for refractory cases of ES based on local availability and the patient's hemodynamic status.
• To evaluate the superiority of propranolol over alternative beta-blockers for the treatment of complex VTs.

## History of Presentation

**Figure 1:**
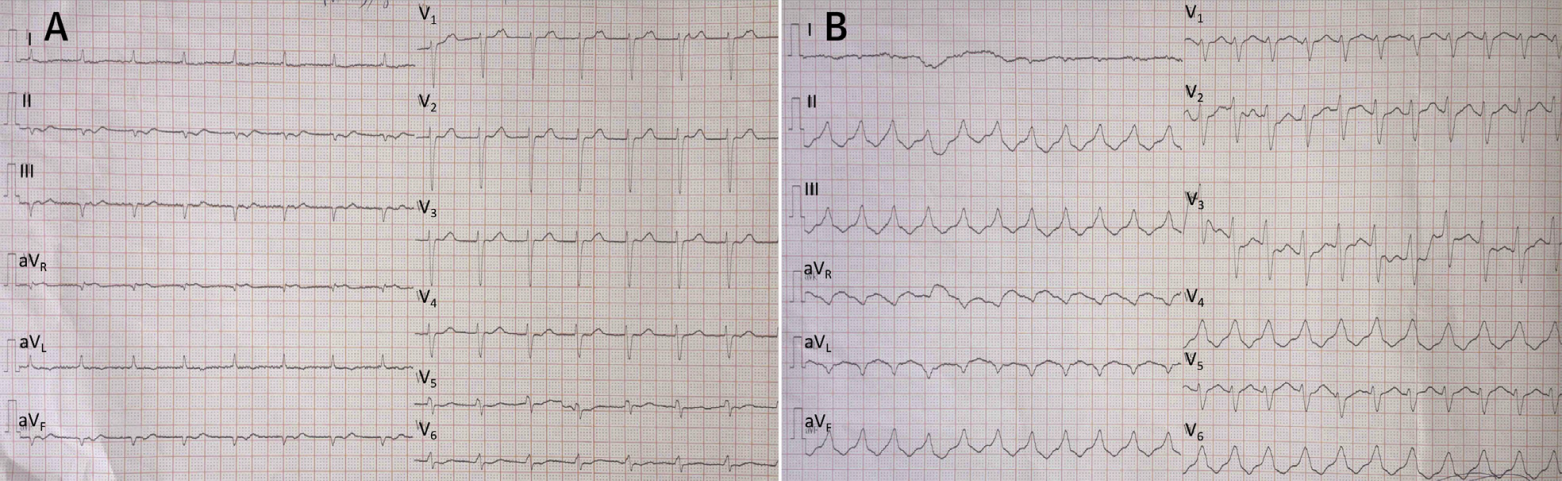
12-Lead ECG The 12-lead electrocardiogram (ECG) of the patient during admission to the intensive care unit (ICU) with **(A)** sinus rhythm and **(B)** ventricular tachycardia.

A 60-year-old man presented to the emergency department due to recurrent ICD-delivered shocks. At hospital arrival, the patient was hemodynamically stable (arterial blood pressure 120/75 mm Hg). Physical examination and electrocardiogram (baseline rhythm: sinus tachycardia, 110 beats/min) (Figure 1) did not provide evidence of acute decompensation of HF or acute coronary syndrome.

## Medical History

The patient's medical history included chronic nonischemic HF (New York Heart Association functional class III), type 2 diabetes mellitus, paroxysmal atrial fibrillation, and dyslipidemia. The device was implanted in 2014 for primary prevention of sudden cardiac death, but no shocks were reported before the index event.

## Differential Diagnosis

Twelve-lead electrocardiography and device interrogation at the emergency department confirmed the presence of multiple episodes of sustained monomorphic VT; thus, the diagnosis of an ES was established. Additional differential diagnoses were not applicable in this case.

## Investigations

**Figure 2:**
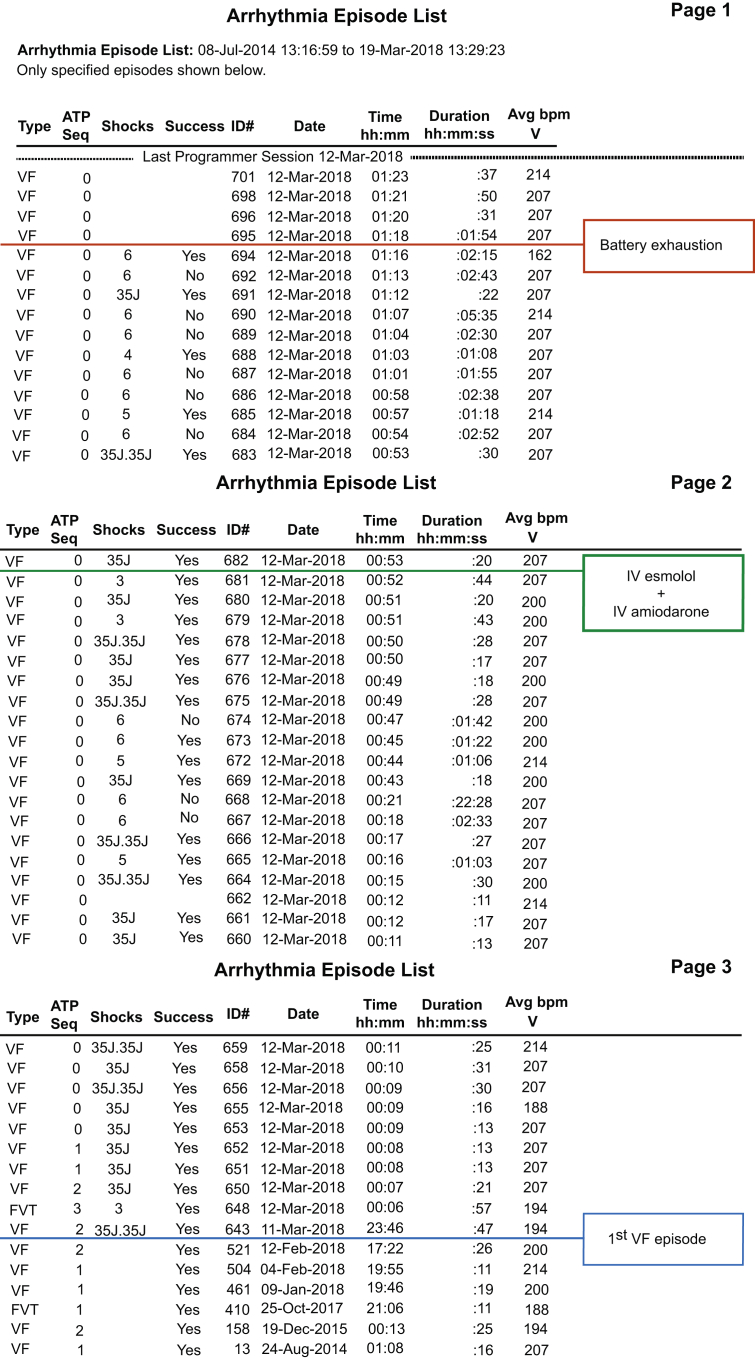
Brief Interrogation Report of Patient’s ICD in the ICU Only the last 3 interrogation strips are displayed, sorted by date. Index shock was delivered on March 11, 2018, at 11:46 pm**(blue box)**. Patient presented to the emergency department at approximately 12:45 am (March 12, 2018). Sixty-nine implantable cardioverter-defibrillator (ICD) discharges were recorded in total until commencement of intravenous (IV) amiodarone and esmolol (12:52 am, annotated by **green box**). Between 12:52 am and 1:16 am, the ICD delivered 55 shocks despite combined antiarrhythmic therapy that was further escalated by adding IV lidocaine, IV magnesium, and IV midazolam. At 1:18 am, the ICD was exhausted (displayed in the **red box** as “battery exhaustion”). Ventricular fibrillation episodes in the **top part of the last strip** (from 1:18 am to 1:23 am) were detected by the device but externally defibrillated. Abbreviation as Figure 1.

Emergent device interrogation was performed, which was compatible with incessant ES. In total, 29 recurrences of ventricular arrhythmias were recorded (28 episodes of VF and 1 episode of VT), treated with 69 discharges and 9 antitachycardia pacing therapies within the last 2 h (Figure 2). Laboratory examinations excluded electrolyte abnormalities, although high-sensitivity troponin I was slightly increased (198 pg/ml). The patient was immediately admitted to the intensive care unit (ICU) under close monitoring.

## Management (Medical/Interventions)

Intravenous (IV) esmolol (bolus infusion rate of 0.5 mg/kg over 1 min, maintenance rate 0.2 mg/kg/min) in combination with IV amiodarone (bolus infusion rate of 30 mg/min over 10 min, maintenance rate 1,000 mg/24 h) was initiated. However, conventional antiarrhythmic therapy failed to abrogate electrical instability, and the patient experienced 17 appropriate shocks within the next hour of ICU hospitalization. Administration of IV magnesium and lidocaine, as well as mild sedation (IV midazolam) on top of the anti-arrhythmic combination did not materially affect the intensity of ES. Ultimately, the ICD power source was exhausted after having delivered 124 shocks in total from the beginning of ES.

Because the patient was hemodynamically stable, the authors opted to switch antiarrhythmic drugs as the next therapeutic approach. In particular, oral administration of propranolol in combination with IV amiodarone led to successful termination of ES. No subsequent ventricular arrhythmias were recorded after a single dose of propranolol (40 mg). Propranolol therapy (20 to 40 mg 4 times a day depending on blood pressure) was retained until day 3 of ICU hospitalization with complete suppression of complex VT/VF arrhythmia.

## Discussion

ES is a life-threatening syndrome associated with dismal short- and long-term prognoses, and its urgent suppression is of vital importance. Beta-blockers are considered the cornerstone of ES management. In a recent study, propranolol on top of IV amiodarone was shown to be superior to selective beta-blockers (metoprolol) in terms of early VT termination and reduction rate of ICD shocks during ICU stay (3).

A growing body of evidence converges on the significant role of the autonomic nervous system in arrhythmogenesis and the pro-arrhythmic properties of elevated circulating levels of catecholamines (4). Patients with chronic HF present with increased baseline sympathetic activity that may precipitate lethal ventricular arrhythmias and sudden cardiac death (5). In this respect, the principal target of ES suppression, either conventionally or invasively, is the sympathetic blockade and the modulation of the autonomic nervous system. To this end, administration of beta-blockers in combination with amiodarone and mild sedation have proved highly effective in the management of ES because they suppress sympathetic tone and increase the VT/VF threshold (6). Propranolol is associated with combined blockade of β1 and β2 receptors. Selective down-regulation of β1 receptors in the failing ventricular myocardium leads to a relatively high proportion of β2 receptors (7). The latter may contribute to the increase in cardiac norepinephrine spillover after administration of selective β1 blockers (8). Moreover, propranolol does not present intrinsic sympathomimetic activity. Therefore, propranolol may more effectively modify cardiac noradrenergic neurotransmission and mitigate reflex sympathetic activation in patients with HF. Propranolol has highly lipophilic properties in pharmacokinetic terms and thus may have a greater central sympathoinhibitory effect (8).

In contrast, interventional procedures are increasingly implemented in refractory forms of ES. Catheter ablation of the VT/VF focal substrate (9,10), stellate ganglionic blockade, and bilateral cardiac sympathetic denervation (10) may reduce the incessant recurrences of ventricular arrhythmia resistant to standard antiarrhythmic treatment and improve short-term outcomes and survival. Recently, renal artery denervation has emerged as a promising adjunctive therapy for refractory VT/VF events in patients with underlying cardiomyopathy (10). Nevertheless, because of the acute presentation of ES and unavailable specialized resources on a local level or 24-h basis, together with potential complications of interventional procedures in such high-risk patients, optimal antiarrhythmic therapy by means of drug switch and/or dose escalation should be always pursued.

Among proposed salvage therapies, general anesthesia might also suppress incessant ventricular arrhythmias (10). However, the potential depression of left ventricular systolic function associated with the use of propofol for inducing and maintaining general anesthesia should be acknowledged, especially in borderline patients who are experiencing ES. In this case, mild sedation with IV benzodiazepine was administered, with the aim of effectively reducing pain and the psychological distress caused by multiple shocks. However, suppression of the arrhythmia burden was not immediately observed. General anesthesia with intubation was suspended and was re-evaluated as an option in the next hours after further discussion with the patient’s family. Whether such an intervention urgently performed could effectively diminish ES cannot be answered; it would also be a reasonable approach.

Finally, propranolol in combination with IV amiodarone was safe and effective in terminating recalcitrant episodes of ventricular arrhythmia and might be preferred to interventional therapy in hemodynamically stable ES in the context of step-by-step management.

## Follow-Up

The clinical course of the patient was complicated on day 5 by an ICU-acquired infection that rapidly decompensated the underlying HF. Despite aggressive management with IV antibiotics, sequential combinations of vasopressors, renal replacement therapy, and mechanical ventilation, the patient died from pump failure on day 24.

## Conclusions

Propranolol in addition to IV amiodarone might be preferred in hemodynamically stable patients with ES before interventional therapies.
